# Spouse and Child Caregivers’ Experiences of Lucid Episodes in Dementia: A Mixed Methods Approach

**DOI:** 10.1093/geroni/igaf036

**Published:** 2025-04-23

**Authors:** Kyungmin Kim, Lauren R Bangerter, Yin Liu, Dawn M Finnie, Maria I Lapid, Joseph E Gaugler, Joan M Griffin

**Affiliations:** Department of Child Development and Family Studies, College of Human Ecology, Seoul National University, Seoul, Korea; Integrated Major in Regional Studies and Spatial Analytics, Seoul National University, Seoul, Korea; Health Economics and Aging Research Institute, MedStar Health Research Institute, Hyattsville, Maryland, USA; Department of Human Development and Family Studies, Utah State University, Logan, Utah, USA; Kern Center for the Science of Healthcare Delivery, Mayo Clinic, Rochester, Minnesota, USA; Department of Psychiatry and Psychology, Mayo Clinic, Rochester, Minnesota, USA; School of Public Health, University of Minnesota, Minneapolis, Minnesota, USA; Kern Center for the Science of Healthcare Delivery, Mayo Clinic, Rochester, Minnesota, USA; Division of Health Care Delivery Research, Mayo Clinic, Rochester, Minnesota, USA

**Keywords:** Appraisals, Dementia grief, Informal caregivers, Paradoxical lucidity

## Abstract

**Background and Objectives:**

Lucid episodes (LEs) in people living with late-stage dementia have been reported anecdotally. However, how this seemingly unexpected phenomenon is experienced by family caregivers is less known. Focusing on the two most common groups of informal caregivers, spouses and adult children, this study examined variability in family caregivers’ experiences with LEs—whether they exhibit differential appraisals of and responses to LEs.

**Research Design and Methods:**

Using a sample of former/bereaved and current family caregivers from UsAgainstAlzheimer’s A-LIST, we conducted an online survey of spouse and child caregivers (*N* = 387). We conducted semistructured interviews among a subset of caregivers who witnessed a LE (*n* = 22).

**Results:**

Overall, child caregivers were more likely to witness a LE than spouse caregivers. Among former/bereaved caregivers who witnessed a LE (*n* = 139), spouses were likely to report nonverbal communication during LEs, appraise LEs more negatively, and make changes in care decisions, such as end-of-life planning and financial decisions, compared to adult children. Among current caregivers who witnessed a LE (*n* = 80), spouses often reported no special circumstances preceding LEs, whereas children linked LEs to friend/family visits. No significant differences were found in positive and negative appraisals of LEs between current spouse and child caregivers. Content analysis of qualitative interviews revealed the contexts underlying these differences.

**Discussion and Implications:**

Differences between spouses and adult children in their experiences with LEs are related to their different caregiving contexts, including relationship history, living arrangements, expectations, motivations, and caregiving resources.

**Translational Significance:**

This study examined variability in family caregivers’ experiences with lucid episodes (LEs) in people living with late stages of dementia. Our findings showed that the caregiver’s relationship to the person living with dementia (i.e., spouse or adult child) and the caregiving status (i.e., former/bereaved or current caregiver) are associated with occurrences, appraisals, interpretations, and perceptions of LEs. In the absence of clinical consensus on LEs, future research is needed to design caregiver services that include support after the death of a person living with dementia, particularly for those caregivers who appraised LEs negatively.

Until recently, lucid episodes (LEs) in people living with advanced stages of Alzheimer’s disease and related dementias (ADRD) had been limited to case reports and anecdotes (Macleod, 2009; Nahm & Greyson, 2009; Normann et al., 2002). These episodes—characterized by spontaneous, unexpected, transient recovery of communication, or other functions among people in the late stages of ADRD who seemingly had a permanent loss in these abilities, are sometimes referred to as *paradoxical lucidity* because of the inconsistency of their occurrence with current pathophysiology models of dementia (Nahm, 2022). A growing body of research shows variation in LE presentation, from how long they last to what kind of communication or function is recovered, and whether they are harbingers for impending death. Previous studies have found that many LEs are brief, with most lasting less than 30 min (Griffin et al., 2024; Karlawish et al., 2024). In some cases, a LE may include changes in communication (e.g., a person with ADRD has not spoken in several years, suddenly turns to his daughter and asks her to turn on the radio so he may listen to the news) or changes in awareness (e.g., a person with ADRD who has not recognized or acknowledged her spouse in several months looks over, waves and smiles when they walk in the door; Ramirez et al., 2024). More recent studies have also begun to describe how this phenomenon is experienced and appraised by family members (Gilmore-Bykovskyi et al., 2023a; Griffin et al., 2022). This line of work suggests a range of decisional and emotional responses after witnessing LEs, including experiences of positive emotion and grief and decisions to modify daily care and treatment plans (Karlawish et al., 2024). The current study adds to the emerging literature by examining variability in family caregivers’ experiences with LEs, focusing on the two most common groups of informal caregivers—spouses and adult children, and whether they may exhibit differential appraisals of and responses to LEs.

Informal dementia caregiving is usually provided in two primary relationships, marriage (or a stable partnership) and adult children caring for aging parents (Wolff et al., 2018). In general, spouses provide more care than adult children, often because they live together (Viñas‐Diez et al., 2017; Wawrziczny et al., 2020). The caregiver’s relationship to the person with ADRD (i.e., spouse vs adult child) is one of the main factors influencing how caregivers appraise their role. Appraisals of caregiving experiences and the grief and burden associated with caring for older adults with ADRD can also vary by relationship (Kim et al., 2012; Pinquart & Sörensen, 2011). Spouses reported less burden than adult children when they transitioned to the caregiving role, characterizing caregiving as a part of their marital role, whereas child caregivers reported more burden due to the significant shift in their role and lifestyle (Conde-Sala et al., 2010). With the gradual, yet enduring loss of cognitive function of the person with ADRD, spousal caregivers could report more burden and feelings of loss than child caregivers (Cheung et al., 2018; Pinquart & Sörensen, 2011). Thus, it is also possible that appraisals of LEs vary by relationship type, although we are unaware of any studies that have sought to understand if spousal caregivers appraise LEs in a different way than child caregivers.

Multiple methodological approaches have been suggested to assess LEs, including informant appraisal, in which an observer, such as a family member, witnesses and reports attributes of the episode (Gilmore‐Bykovskyi et al., 2023b), and to characterize LEs. In our previous work, we developed a typology of LEs identifying four potential types of LEs, using quantitative survey data (Griffin et al., 2024). Understanding if there is variability in family caregivers’ experiences of LEs can help to improve measurement in this emerging area of research (Kim & Knight, 2018; Montgomery & Kosloski, 2013). Specifically, considering subgroup differences in interpreting LEs can help tailor interventions to support caregivers cope with LEs. The current study focuses on the two most common groups of informal caregivers, spouses and adult children, and examines whether they exhibit differential appraisals of and responses to LEs.

## Method

### Data and Sample

**Figure 1.**
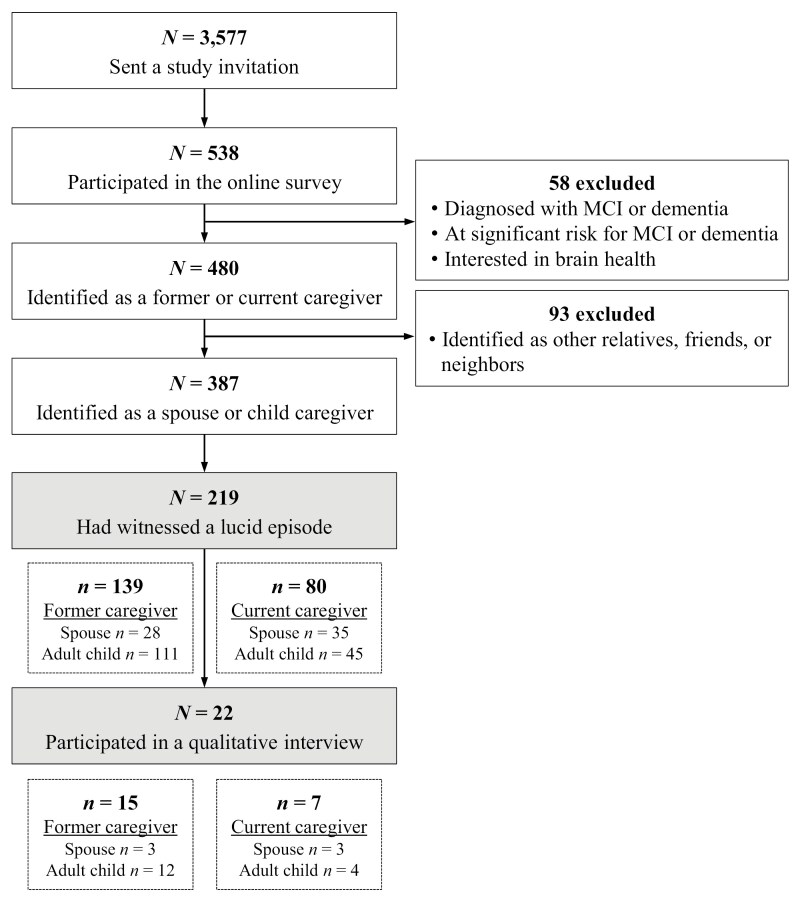
Flow Chart of the Sample Selection.

This study is based on data collected using a sequential explanatory design, where the quantitative data were collected first, followed by qualitative data (Creswell et al., 2003; see Figure 1 for the flow chart of sample selection). This study recruited former/bereaved or current caregivers of people living with ADRD from the A-LIST (administered by UsAgainstAlzheimer’s), which is a unique online community interested in participating in research on dementia. An email invitation with a hyperlink to an electronic survey was sent to 3,577 participants of the A-LIST who had either identified as a former/bereaved or current caregiver or had not specified their role in dementia caregiving. UsAgainstAlzheimer’s Institutional Review Board (IRB) approved the study and all participants provided informed consent prior to participating.

A total of 538 people participated in the online survey that included questions about their experience of LEs, caregiving contexts, and background characteristics; 58 (11%) were not eligible for the study (i.e., not a former/bereaved or current caregiver). Out of the 480 eligible participants, 93 caregivers had other relationship types (i.e., friends or neighbors) to the person with ADRD rather than spouses and adult children and were excluded from the present analysis. The analytic sample was 387 participants (144 spouse caregivers and 243 child caregivers).

Among caregivers who indicated their willingness to participate in a follow-up interview about their LE experiences in the online survey (179 out of 219; see Figure 1), 22 participated in an in-depth interview via telephone (6 spouse caregivers and 16 child caregivers; see Supplementary Table 1 in Supplementary Material for sample characteristics). Interview participants were purposively sampled based on the type of LEs they witnessed, and then, when possible, balanced to represent relationships to the older adult with ADRD (i.e., spouse vs others), coresidence status (i.e., living together vs not living together), and whether the older adult with ADRD was still living or had died. The Mayo Clinic IRB approved the interview protocol, and participants provided oral consent prior to the interview.

### Measures

#### Survey measures

The survey provided a general description of a LE: “A lucid experience is an unexpected, spontaneous, meaningful, and relevant communication with your relative, friend, or neighbor when they had lost the ability to speak or have personal interactions.” Participants were asked if they had ever witnessed a LE in their relative with ADRD (1 = *yes*, 0 = *no*). If participants answered yes, they reported on characteristics and details of the most memorable LE witnessed up to two episodes (Batthyány & Greyson, 2021; Griffin et al., 2024). Characteristics of LEs included (a) the relative’s cognitive status on a typical day before the LE (with six response options: 1 = *fully aware with no impairments*; 2 = *minor difficulty with memory, attention, or focus*; 3 = *moderate difficulty with memory, attention, or focus*; 4 = *extreme difficulty with memory, attention, or focus*; 5 = *awake, but not responding or reacting*; 6 = *mostly asleep/unconscious*), (b) the relative’s proximity to death (with six response options: 1 = *still alive*; 2 = *lived more than 6 months*; 3 = *1 week–6 months before death*; 4 = *4–7 days before death*; 5 = *2–3 days before death*; 6 = *within 24 hr of death*), (c) duration of LEs (with seven response options: 1 = *under 10 min*; 2 = *11–30 min*; 3 = *31–60 min*; 4 = *1–4 hr*; 5 = *4–24 hr*; 6 = *2–7 days*; 7 = *more than 7 days*), (d) communication quality (with five response options: 1 = *aware, communication made complete sense*; 2 = *aware, communication made some sense*; 3 = *aware, but communication made no sense*; 4 = *aware, but only nonverbal communication*; 5 = *talked coherently while sleeping*), and (e) circumstances prior to LEs (e.g., visits from family or friends, change in medication, change in healthcare setting, and reminiscing; 1 = *yes*, 0 = *no*).

To assess how caregivers responded to the LEs, we included (a) emotional appraisals of LEs (i.e., how positive or stressful it was; rated from 1 = *not at all* to 5 = *very*), (b) behavioral changes in care patterns or decisions after LEs (e.g., medical care, finances, end-of-life planning, and living arrangements; 1 = *yes*, 0 = *no*), and (c) information seeking after LEs (e.g., whether and from whom they sought out information or education about LEs; 1 = *yes*, 0 = *no*).

We also considered demographic characteristics of caregivers and care receivers for caregiving contexts, including the dyads’ age, gender, education, race/ethnicity, marital status, work status, coresidence status, and dementia type.

#### Qualitative interviews

The semistructured interview guide was designed to gain a holistic, in-depth understanding of the experiences of LEs from the caregivers’ perspective. As an extension of the quantitative survey questions, the qualitative interviews began with questions to further elicit general information about the relationship between the caregiver and the person with ADRD, as well as qualities and personality traits of the person with ADRD. Next, we asked questions about the LE that the caregiver witnessed, including probes about special circumstances, whether the person with ADRD was aware of the lucid moments, and other circumstances that distinguished the LE from normative cognitive fluctuation or “good days or bad days.” We then asked questions about what the experience of the LE was like for the caregiver and whether or not the LE changed decisions that the caregiver made for the person with ADRD. We concluded the interview by asking about the lasting impression of the LE as well as the proximity to the death of relatives with ADRD (e.g., as a stressful or positive experience; see Supplementary Material for interview questions).

### Analysis

To obtain enough LE reports among dementia caregivers, the analytic sample included both former/bereaved caregivers whose relatives with ADRD had died by the time of the survey and current caregivers with living relatives with ADRD. The nature of LE reports (e.g., bereaved caregivers’ reports may be more distal and retrospective than current caregivers’ ones) and caregiving contexts (e.g., current caregivers’ care responsibilities are ongoing, whereas bereaved caregivers no longer provide care for the relative with ADRD) may be qualitatively distinctive between former and current caregivers (Stahl & Schulz, 2019). Therefore, we further stratified spouse-child comparisons by care receivers’ living status (139 former/bereaved caregivers [28 spouses vs 111 children]; 80 current caregivers [35 spouses vs 45 children]). Using bivariate statistical analyses (i.e., *t* or *χ*^2^ tests), all caregivers’ experiences and responses to LEs as well as demographic characteristics were compared between spouses and adult children, separately for former/bereaved and current caregivers. Also, we estimated multivariate models stratified by care receivers’ living status (i.e., former/bereaved vs current caregivers) to examine predictors for emotional appraisals to LEs, including the relationship type (i.e., spouse vs child caregivers) as well as other characteristics. The clustered nature of the data (up to two episodes per participant) was handled using generalized estimating equations.

Qualitative data were analyzed using the following IRB-approved approach. Telephone interviews were digitally recorded and transcribed verbatim. Our approach to the narrative interview data was to identify recurrent themes and define categories that reflected these themes (Hsieh & Shannon, 2005). Specifically, the primary analyst (D. M. Finnie) used open-coding, where codes and their corresponding definitions were first developed from review of transcripts and then refined with subsequent review of transcripts. After the first 25% of the interviews were completed, two other study investigators (J. M. Griffin and L. R. Bangerter) reviewed and discussed codes and then refined both the codebook and the interview guide. This same process was repeated after the next 25% of interviews were done—until there was consensus on definitions of codes and the codebook was considered final. Codes were then assigned, line-by-line, to the transcripts. Coded data were then analyzed to identify key themes around how spouses and adult children appraised LEs.

## Results

### Survey Findings on Spouse and Child Caregivers’ Experiences of Lucid Episodes

Of the 387 eligible participants who were identified as (former/bereaved or current) spousal or child caregivers, 56.8% reported witnessing a LE at any time. Adult children were more likely to report a LE than spouses (65% vs 44%; not shown in table). We limited our main analysis to those caregivers who reported witnessing a LE (*N* = 219) to compare their LE experiences and responses (episode *N* = 412; 193 reported two episodes and 26 reported one episode) between spousal and child caregivers.

Table 1 displays characteristics of caregivers and their relatives with ADRD. As expected, spouses were older and more likely to live with their relative with ADRD in the same household than adult children among both former/bereaved and current caregivers. Among current caregivers, spouses were more likely to be non-Hispanic White; they were also less likely to be employed than adult children. Regarding dementia types, former/bereaved spousal caregivers were more likely to report that their relative was diagnosed with Frontotemporal dementia or Lewy body dementia; current child caregivers were more likely to report that their relative was not formally diagnosed.

**Table 1.** Characteristics of Family Caregivers and Their Relatives With Dementia

Variables	Total	Former caregiver		Current caregiver	
		Spouse	Adult child	Spouse	Adult child
Participant *N*	219	28	111	35	45
Episode *N*	412	53	205	67	87
**Caregivers**					
Age^a^, *M* (*SD*)	5.02 (1.02)	5.68^*^ (0.77)	4.93 (0.89)	5.83^*^ (0.89)	4.20 (0.84)
Education^b^, *M* (*SD*)	4.66 (1.41)	4.32 (1.36)	4.82 (1.40)	4.63 (1.54)	4.49 (1.36)
Female, %	79	72	81	73	82
White, %	86	89	89	94^*^	71
Married, %	64	21	65^*^	97^*^	64
Employed, %	29	25	32	3	44^*^
Coresiding with care receiver, %	43	71^*^	23	83^*^	42
**Relatives with dementia**					
Age^c^, *M* (*SD*)	83.10 (9.16)	76.22 (11.93)	86.22^*^ (7.26)	77.21 (7.85)	86.27^*^ (7.39)
Education^b^, *M* (*SD*)	3.31 (1.67)	3.93^*^ (1.78)	2.80 (1.50)	4.31^*^ (1.57)	3.39 (1.62)
Female, %	63	29	68^*^	40	91^*^
White, %	87	93	90	94^*^	71
Dementia type^d^, %					
Alzheimer’s disease	71	71	77	71	56
Vascular	11	11	10	11	16
Frontotemporal	8	21^*^	3	11	9
Lewy body	6	18^*^	5	3	2
Parkinson	2	7	2	3	0
Never diagnosed/don’t know	10	0	9	6	22^*^

*Notes*: Participant *N* = 219 (episode *N* = 412).

^a^1 = *under age 30*, 2 = *age 31–40*, 3 = *age 41–50*, 4 = *age 51–60*, 5 = *age 61–70*, 6 = *age 71–80*, 7 = *age 81–90*, and 8 = *over age 90*.

^b^1 = *some primary or high school*, 2 = *high school or equivalent*, 3 = *vocational or technical degree*, 4 = *associate’s degree*, 5 = *bachelor’s degree*, 6 = *master’s degree*, and 7 = *doctoral degree*.

^c^Age at death for former/bereaved caregivers; current age for current caregivers.

^d^Selected all response options that apply.

^*^Indicating a significant difference between spouses and adult children (*p* < .05), based on *t* or *χ*^2^ tests.

Table 2 summarizes the characteristics of the most memorable LEs that caregivers witnessed—up to two episodes. Among former/bereaved caregivers, spouses were more likely to report that the LEs occurred as nonverbal communication (19% vs 7%), compared to adult children. Among current caregivers, adult children were more likely to report that the LEs lasted 31–60 min (10% vs 2%) than spouses. Also, spouses were more likely to indicate no special circumstances prior to LEs (57% vs 33%), whereas adult children were more likely to indicate LEs being associated with visits from family or friends (44% vs 19%).

**Table 2.** Characteristics of Lucid Episodes

Variables	Total	Former caregiver		Current caregiver	
		Spouse	Adult child	Spouse	Adult child
**Cognitive status**, %					
Fully aware with no impairments	2	2	2	1	2
Minor difficulty	4	4	3	7	6
Moderate difficulty	26	13	23	31	36
Extreme difficulty	44	43	47	45	38
Awake, but not responding or reacting	14	23	13	13	13
Mostly asleep/unconscious	9	15	11	1	6
**Proximity to death**, %					
Still alive	39	6	2	98	98
Lived more than 6 months	30	38	49	2	2
1 week to 6 months before death	20	34	33	0	0
4–7 days before death	5	10	7	0	0
2–3 days before death	3	4	4	0	0
Within 24 hr of death	3	8	4	0	0
**Duration**, %					
Under 10 min	56	47	53	66	61
11–30 min	21	27	25	19	10
31–60 min	9	16	8	2	10^*^
1–4 hr	7	6	7	3	9
4–24 hr	4	0	4	6	4
2–7 days	1	2	1	0	3
More than 7 days	2	2	2	3	4
**Communication quality**, %					
Aware, communication made complete sense	51	47	51	58	48
Aware, communication made some sense	34	26	35	29	40
Aware, but communication made no sense	6	8	7	5	5
Aware, but only nonverbal communication	9	19^*^	7	6	7
Talked coherently while sleeping	0	0	0	3	0
**Circumstances prior to lucid episodes** ^a^, %					
No special circumstances	36	38	29	57^*^	33
Visits from family/friends	35	32	38	19	44^*^
Change in medication	5	6	2	4	11
Change in healthcare setting	7	9	10	3	1
Music playing that was meaningful to them	14	11	15	16	11
Reminiscing (e.g., looking at pictures)	8	6	9	6	11
Value rituals/behavior (e.g., religious activity)	5	2	7	4	3

*Notes*: Participant *N* = 219 (episode *N* = 412).

^a^Selected all response options that apply.

^*^Indicating a significant difference between spouses and adult children (*p* < .05), based on *χ*^2^ tests.

We also compared caregivers’ emotional and behavioral responses to LEs (see Table 3). Positive appraisals of LEs were higher than negative appraisals of LEs across all types of caregivers. Among former/bereaved caregivers, spouses were likely to appraise LEs more negatively and make ensuing changes in care decisions (i.e., end-of-life planning and financial decisions), compared to adult children. However, there was no difference in emotional appraisals of LEs and behavioral responses (e.g., changes in care decisions and information seeking) between current spouse and child caregivers.

**Table 3.** Family Caregivers’ Responses to Lucid Episodes

Variables	Total	Former caregiver		Current caregiver	
		Spouse	Adult child	Spouse	Adult child
**Emotional appraisal** ^a^, *M* (*SD*)					
Positive	4.01 (1.26)	4.09 (1.20)	4.05 (1.25)	3.87 (1.19)	3.98 (1.36)
Negative	1.96 (1.33)	2.58^*^ (1.53)	1.93 (1.23)	1.73 (1.33)	1.83 (1.34)
**Any decision changes** ^b^, %	13	11	8	17	22
Medical care	5	4	2	6	16
Finances	1	4^*^	0	3	0
End-of-life planning	2	7^*^	0	0	7
Living arrangements	6	0	5	11	11
Personal needs	5	4	1	6	13
Social needs	5	7	5	6	7
How to provide care	4	4	2	6	9
**Seeking out information** ^b^, %	13	14	13	14	13
From healthcare providers	8	7	9	6	9
From internet	8	14	8	9	4
From family/friends	2	0	5	0	0
From public educators/media	1	4	1	0	0
From support group	0	0	0	0	2
From Alzheimer’s Association	0	0	1	0	0

*Notes*: Participant *N* = 219 (episode *N* = 412).

^a^1 = *not at all* to 5 = *very*.

^b^Selected all response options that apply.

^*^Indicating a significant difference between spouses and adult children (*p* < .05), based on *t* or *χ*^2^ tests.

Finally, we conducted multivariate analyses to confirm whether the bivariate differences observed in the emotional appraisals of LEs between spouses and adult children would remain after adjusting for spouse–child differences in other characteristics (see Table 4). The results showed that former/bereaved spouse caregivers were likely to appraise LEs more negatively than child caregivers, even after controlling for other characteristics. Current caregivers’ emotional appraisals of LEs did not differ by the relationship type. However, coresidence was a more significant factor in emotional appraisals of LEs; caregivers living in the same household with their relative with ADRD were likely to appraise LEs more negatively and less positively.

**Table 4.** Multivariate Models for Family Caregivers’ Emotional Appraisals of Lucid Episodes

Variables	Former caregiver				Current caregiver			
	Positive appraisal		Negative appraisal		Positive appraisal		Negative appraisal	
	*B*	(*SE*)	*B*	(*SE*)	*B*	(*SE*)	*B*	(*SE*)
Spouse	0.31	(0.25)	0.97**	(0.33)	0.00	(0.49)	−0.00	(0.42)
***Controls***								
Coresiding	−0.03	(0.25)	−0.22	(0.22)	−0.92**	(0.32)	0.76**	(0.28)
Female	−0.11	(0.24)	−0.07	(0.26)	−0.18	(0.27)	0.22	(0.27)
Age	−0.08	(0.13)	−0.19	(0.12)	0.17	(0.18)	−0.20	(0.17)
Education	0.01	(0.07)	−0.02	(0.07)	−0.06	(0.10)	−0.22^*^	(0.09)
White	−0.30	(0.31)	0.21	(0.29)	−0.26	(0.44)	0.32	(0.25)
Quasi-likelihood	352.59		389.32		205.89		181.71	

*Notes*: Former caregiver *n* = 139 (episode *n* = 258); current caregiver *n* = 80 (episode *n* = 154). The clustered nature of the data (up to two episodes per participant) was handled using generalized estimating equations (GEE).

^*^ *p < *.05. **p < .01.

### Qualitative Analysis of Caregivers’ Experiences of Lucid Episodes

Content analysis of interviews revealed three themes connected to the quantitative findings on caregiver responses to and emotional appraisals of LEs. The first theme “*Ambiguity around LEs*” captures the complexity of appraising LEs. Caregivers in our study experienced significant uncertainty about LEs, as one caregiver describes (former spouse caregiver): “*The [spouse with ADRD] regressed again immediately. I mean I visited the next day or the day after and vegetable. So, even at the time, I think I did know that this happened. I never saw it as a turnaround, no, just a mystery*.” Often, caregivers who witnessed multiple LEs changed their appraisals of LEs over time. Some caregivers described how LEs contributed to a sense of hope that the relative with ADRD was going to regain cognitive abilities. However, after repeated exposure to LEs, this sense of hope became less prominent (current child caregiver): “*Up until probably about five years ago, I was pretty hopeful. They were doing research on Alzheimer’s and stuff like that. But now, at this point, she’s on hospice five out of seven days a week. So, [dying] not a matter of if. It’s just a matter of when. So, I’m no longer hopeful at all*.”

The second theme “*Gratitude for LEs*” captures the important contextual factors that contribute to positive appraisals of LEs. These findings provided important contexts for how and why caregivers felt gratitude for LEs. One caregiver explained that they had feelings of guilt with regard to their father’s quality of life and well-being as a person living with ADRD, but a LE in which her father discussed significant memories helped to alleviate these feelings (former child caregiver): “*It was a gift… He was remembering his life in front of us, and we were able to witness that, you know, and not have to be stuck with the thought of this man on morphine because he had broken his back because he fell out of bed in the nursing home... It was totally wonderful for us*.” For another caregiver, witnessing a LE where the relative with ADRD was joyful and happy had a similar impact and helped the caregiver reconcile with the reality that the relative with ADRD was institutionalized (current child caregiver): “*And [mother with ADRD] just was just like a kid. It was like, oh my God! She’s having so much fun. She’s really enjoying herself and just. She just sparked up and was just her old self again. It was amazing… to see her and know that she was enjoying herself because I always felt guilty with her being in [Nursing Home]*.”

The third theme “*Negative Appraisal of LEs*” captures the experience of caregivers who viewed LEs as particularly negative and harmful to their well-being. Caregivers explained their disappointment when they failed to recreate LEs for the relative with ADRD, noting that the brevity and unpredictability of LEs were particularly frustrating. One caregiver describes the devastating experience of a relative with ADRD demonstrating full awareness of the burden and challenges that their dementia diagnosis had on others (former child caregiver): “*She undid her seatbelt, and she crept down into the front of the car, like on the floor, in the fetal position, and she said, ‘I know that this is really hard for you, and I know this is going to be much harder for you than it’s going to be for me, and I’m so sorry.’ And I just, I mean, I had to pull over. Uhhh, I just had to pull over because it was horrible for me because just having your mom apologizing for being sick was just brutal.*” Another caregiver explained that they were still, years later, trying to cope with the lasting impact of LEs on their own mental well-being (current spouse caregiver): “*I just could not figure out why, like what happened, like what made [LE] happen. So, I wish, for people that have to have their [relative with ADRD] be lucid and stuff, I wish that I could shed some advice and tell them that it is a blessing or it is a gift, but I just found it to be very, very hard for me*.”

## Discussion

The LEs in dementia remain an unpredictable, under-recognized, and clinically unclear phenomenon. However, findings from the current study highlight some of the most important nuances of caregiver’s appraisal of LEs. Utilizing the mixed methods approach, this study aimed to investigate how family caregivers—specifically, spouses and adult children, experience and appraise LEs in people living with advanced stages of dementia. We explored whether these groups exhibit different appraisals of LEs, differences in their emotional and behavioral responses, and the contextual factors that contribute to these differences.

LEs were appraised differently by spouses and adult children in our study, which aligns with demonstrated differences in the experiences and emotional and financial burdens of spousal and adult child caregivers (Fenton et al., 2022; Oldenkamp et al., 2016). The caregiver’s relationship to the person living with ADRD may contribute to different interpretations of cognitive and behavioral symptoms of dementia, including LEs (Huertas-Domingo et al., 2023). Adult children were more likely to report the occurrence of LEs, which may indicate that these episodes were more apparent, unique, or memorable when witnessed by adult children. Research has demonstrated that child caregivers may see their relative with ADRD less frequently than spousal caregivers (Rigby et al., 2019), which could explain why LEs were more memorable and therefore reported more often by adult children. Among former/bereaved caregivers who witnessed a LE, however, spouses were more likely to report nonverbal communication during the LEs than adult children; their long relationship history may enable to detect lucid moments through nonverbal actions easily.

One of the most pressing questions about LEs is whether there are identifiable triggers, events, or circumstances that precede or cause LEs. This is a question whose answer has yet to reach clinical consensus (Bangerter et al., 2024). Our study found that perspectives on what preceded LEs varied by relationship type and caregiving status. Among current caregivers, adult children were more likely to report that visits from family and friends preceded the LE than spouses who were more likely to report that no special circumstances preceded LEs. This finding indicates that caregiver reports may not be enough to understand and identify predictors of LEs; rather, there is a need for additional data (e.g., biological markers and actigraphy data) to understand changes in physiology that could contribute to LEs (Gilmore‐Bykovskyi et al., 2023b). Thus, a potential next step to advance the scientific understanding of LEs is to incorporate physiological measures and remote monitoring to learn more about potential physiological changes that may be associated with LEs (David et al., 2023).

Caregivers in our study also appraised these LEs differently based on whether they were a former/bereaved or current caregiver. Former/bereaved spousal caregivers tended to have a more negative appraisal of LEs. The unpredictable and seemingly random nature of LEs may interrupt a previously predictable and steady trajectory of cognitive declines, leading to a negative appraisal in retrospect (Cheung et al., 2018). This was evident in our qualitative interviews. The finding that former/bereaved spousal caregivers recalled LEs more negatively than current caregivers may also indicate a need for enhanced support for former/bereaved caregivers. This is important because most caregiver support services are targeted at current caregivers, but our findings suggest that LEs are linked to longer-term negative appraisals that warrant enhanced caregiver support even after the person with ADRD has died. More work is needed to design caregiver services that include support after the death of a person with ADRD (Yeh et al., 2021), particularly for those caregivers who appraised LEs negatively.

The small body of research on LEs has primarily focused on LEs as a phenomenon that occurs near the death of the person with ADRD. Our findings on family experiences have important applications to the end-of-life experience of people in the late stages of ADRD and informal caregivers (Lindgren et al., 1999). Although grief is not a focus of the current study, there is a high prevalence of grief in dementia caregivers before the physical death of the person with ADRD (Lindauer & Harvath, 2014). The unique aspect of dementia grief is that caregivers experience predeath grief akin to post-death bereavement even though the person with ADRD is still alive (Sanders et al., 2008). This is relevant to our study findings because most LEs in our study occurred within 6 months of the care receiver’s death. LEs can exacerbate the ambiguity of dementia grief, in perhaps the most extreme way, by showing a brief yet profound change in cognition. In some cases, LEs may force caregivers to adapt to this sudden cognitive change (as is evident by our finding that former/bereaved spousal caregivers were more likely to make changes in care following a LE). In the absence of clinical consensus on LEs, there are few resources for caregivers to effectively understand how LEs play into the overall trajectory of dementia history, or what changes in clinical decisions could be made when LEs occur.

A key finding from our qualitative interviews is that LEs are often an ambiguous experience. One factor adding to this ambiguity is the fact that few resources and information exist to educate caregivers on LEs. Caregivers have few answers as to why, how, and when LEs occur and what these events mean for the person with ADRD. Caregivers in our study described feeling hope, and then feeling let down when LEs stopped occurring. This false hope can contribute to negative appraisals of LEs and likely affects caregiver well-being.

Our study findings should be interpreted within the context of several limitations. First, our analysis focused on two types of caregivers, spouses and adult children. This is a major limitation because caregiving is often provided by a range of individuals outside of these relationships, including other family members, friends, and neighbors. Thus, our results only captured a limited perspective and future research should seek to have a broader definition of caregiving than we used in the present study. Second, our analyses used a relatively small and selective sample. Compared to national samples of family dementia caregivers (Patterson et al., 2023), our sample included more spouses, females, and non-Hispanic white, but fewer employed and coresiding caregivers. Findings may not be generalizable to a broader caregiving population. Future work should seek to conduct studies on LEs with larger and more racially/ethnically diverse samples and to collect data longitudinally so that changes and patterns in LEs can be readily identified. Finally, given the early stage of current LE research, the retrospective recall approach may be appropriate, but this methodology is often associated with bias and errors. Future studies need to consider prospective/longitudinal designs to reduce recall bias and capture caregivers’ concurrent experiences with LEs.

Despite these limitations, our study has contributed some valuable perspectives to the emerging body of work around LEs and has illuminated some key areas where future research should focus, including advancing the measurement of LEs to incorporate data beyond caregiver reports. This study illuminates some important nuances of LEs that were not previously known and has informed valuable next steps to advance the research agenda in this area.

In conclusion, findings from the current study highlight some of the most important nuances of caregiver’s appraisal of LEs. Utilizing the mixed methods approach, the study showed how the caregiver’s relationship to the person with ADRD (e.g., spouse or adult child) and the caregiving status (e.g., former/bereaved or current caregiver) are associated with occurrences, appraisals, interpretations, and perceptions of LEs. Our qualitative findings offer important insights into caregiving contexts for caregivers’ differential appraisals, highlighting intriguing nuances around ambiguity, gratitude, and negative views of LEs. Future research should continue to explore these dynamics with larger and more diverse samples to enhance our understanding of LEs and improve caregiver support.

## Supplementary Material

igaf036_suppl_Supplementary_Materials_1
