# Supplementary material for: Spouse and Child Caregivers’ Experiences of Lucid Episodes in Dementia: A Mixed Methods Approach
Source: Innov Aging. 2025 Apr 23;9(6):igaf036. doi: 10.1093/geroni/igaf036 (PMC12149523; doi:10.1093/geroni/igaf036)
Supplement: igaf036_suppl_Supplementary_Materials_1 [file igaf036_suppl_supplementary_materials_1.docx]

***Innovation in Aging* Supplementary Material: Kim et al. Spouse and Child Caregivers’ Experiences of** **Lucid Episodes in Dementia: A Mixed Methods Approach.**

**Supplementary Table 1.** *Characteristics of the Qualitative Sample*

| No | Relationship to  care receiver | Coresidence status | Care receiver  living status | Care receiver  gender | Caregiver  gender |
| --- | --- | --- | --- | --- | --- |
| 1 | Spouse | No | Still living | Male | Female |
| 2 | Adult child | No | Died | Male | Female |
| 3 | Adult child | No | Died | Female | Female |
| 4 | Spouse | No | Died | Male | Female |
| 5 | Adult child | No | Still living | Female | Female |
| 6 | Adult child | No | Still living | Female | Female |
| 7 | Adult child | No | Died | Female | Female |
| 8 | Adult child | No | Died | Male | Female |
| 9 | Adult child | Yes | Still living | Female | Female |
| 10 | Adult child | No | Died | Male | Female |
| 11 | Adult child | Yes | Died | Female | Female |
| 12 | Spouse | Yes | Died | Male | Female |
| 13 | Adult child | No | Died | Female | Female |
| 14 | Spouse | Yes | Still living | Male | Male |
| 15 | Adult child | Yes | Still living | Female | Female |
| 16 | Spouse | Yes | Died | Male | Male |
| 17 | Adult child | No | Died | Female | Female |
| 18 | Adult child | No | Died | Male | Female |
| 19 | Adult child | No | Died | Female | Female |
| 20 | Spouse | Yes | Still living | Male | Female |
| 21 | Adult child | No | Died | Male | Female |
| 22 | Adult child | No | Died | Female | Female |

*Notes*. *N* = 22 (6 spouses and 16 adult children).

**Supplementary Section**

*Caregiver Interview Questions*

| 1. Tell me about yourself and your relationship with your loved one. | |
| --- | --- |
|  | *a. Probe: Length of relationship; proximity to living together; knowledge of history* |
| 1. How would you describe your loved one? What were some of the qualities you remember best? | |
|  | *a. Probe: Talkative; outgoing, etc.* |
| 1. Can you tell me about of their illness (Alzheimer’s disease and related dementias), what did you begin to notice and when? | |
|  | *a. Probe: Changes in behavior; changes in affect* |
| 1. You described lucid episodes that you witnessed in the survey that we had sent out. Would you talk about that episode and what you witnessed? | |
|  | *a. Probe: Special circumstances; changes in communication; changes in movement*  *b. Probe: Degree of change; type of communication; self-awareness of lucidity*  *c. Probe: Other circumstances that distinguish from delirium* |
| 1. What was that experience like for you as a caregiver? | |
| 1. How did the lucid episode change decisions you make for the person with dementia? | |
| 1. If the lucid episode was close to death, can you describe him/her before the lucid episode and after? | |
| 1. If the lucid episode was not close to death, can you describe how the episode was different from are sometimes referred to as “good days or bad days”? | |
| 1. What are your lasting impressions of the lucid episode? Was it stressful, confusing, or positive? | |
